# HIV-1 IN/Pol recruits LEDGF/p75 into viral particles

**DOI:** 10.1186/s12977-014-0134-4

**Published:** 2015-02-12

**Authors:** Belete Ayele Desimmie, Caroline Weydert, Rik Schrijvers, Sofie Vets, Jonas Demeulemeester, Paul Proost, Igor Paron, Jan De Rijck, Jan Mast, Norbert Bannert, Rik Gijsbers, Frauke Christ, Zeger Debyser

**Affiliations:** Department of Pharmaceutical and Pharmacological Sciences, KU Leuven, Laboratory for Molecular Virology and Gene Therapy, Leuven, Flanders Belgium; KU Leuven, Laboratory of Molecular Immunology, Rega Institute, Leuven, Flanders Belgium; Department of Proteomics and Signal Transduction, Max-Planck Institute of Biochemistry, D-82152 Martinsried, Germany; Veterinary and Agrochemical Research Centre CODA-CERVA, Brussels, Belgium; Robert Koch Institute, Centre for HIV and Retrovirology, Berlin, Germany; Present address: Viral Mutation Section, HIV Drug Resistance Program, Center for Cancer Research, National Cancer Institute, Frederick, MD USA

**Keywords:** Integrase, LEDGF/p75, Protease, Protease cleavage sites, Assembly

## Abstract

**Background:**

The dynamic interaction between HIV and its host governs the replication of the virus and the study of the virus-host interplay is key to understand the viral lifecycle. The host factor lens epithelium-derived growth factor (LEDGF/p75) tethers the HIV preintegration complex to the chromatin through a direct interaction with integrase (IN). Small molecules that bind the LEDGF/p75 binding pocket of the HIV IN dimer (LEDGINs) block HIV replication through a multimodal mechanism impacting early and late stage replication including HIV maturation. Furthermore, LEDGF/p75 has been identified as a Pol interaction partner. This raised the question whether LEDGF/p75 besides acting as a molecular tether in the target cell, also affects late steps of HIV replication.

**Results:**

LEDGF/p75 is recruited into HIV-1 particles through direct interaction with the viral IN (or Pol polyprotein) and is a substrate for HIV-1 protease. Incubation in the presence of HIV-1 protease inhibitors resulted in detection of full-length LEDGF/p75 in purified viral particles. We also demonstrate that inhibition of LEDGF/p75-IN interaction by specific mutants or LEDGINs precludes incorporation of LEDGF/p75 in virions, underscoring the specificity of the uptake. LEDGF/p75 depletion did however not result in altered LEDGIN potency.

**Conclusion:**

Together, these results provide evidence for an IN/Pol mediated uptake of LEDGF/p75 in viral particles and a specific cleavage by HIV protease. Understanding of the possible role of LEDGF/p75 or its cleavage fragments in the viral particle awaits further experimentation.

**Electronic supplementary material:**

The online version of this article (doi:10.1186/s12977-014-0134-4) contains supplementary material, which is available to authorized users.

## Background

Replication of the human immunodeficiency virus type-1 (HIV-1) is characterized by a dynamic interplay between the virus and the infected cell with a specific temporal and spatial regulation of the virus-host protein-protein interaction (PPI) network. Integration of lentiviral genomic DNA into host chromatin is directed by the viral integrase (IN) and the cellular cofactor lens epithelium-derived growth factor (LEDGF/p75) [1-5], a transcriptional co-activator that tethers a variety of proteins to the chromatin [6-10]. Whereas the N-terminal PWWP domain of LEDGF/p75 reads chromatin modifications [11-13], the C-terminal integrase binding domain (IBD) interacts with lentiviral IN [14]; together they direct integration into active transcription units [2,15-18]. As a molecular tether and a targeting determinant, LEDGF/p75 is recognized as a crucial cellular cofactor of HIV infection, although the related Hepatoma-derived growth factor Related-Protein 2 (HRP-2) can substitute for LEDGF/p75 in its absence [17,19,20]. Additionally, LEDGF/p75 has been shown to interact with the HIV-1 Pol precursor protein in Jurkat cells [21].

In 2010 structure-based drug design has led to the discovery of small molecules that bind in the LEDGF/p75 binding pocket of the IN catalytic core dimer and inhibit HIV replication [22], referred to as LEDGINs (small molecules binding to the LEDGF/p75 binding pocket of integrase). Similar molecules were later reported by other groups [23-27]. LEDGINs disrupt the interaction of IN with LEDGF/p75 and act as allosteric IN inhibitors. The latter mechanism of action is attributed to enhanced IN multimerization [23-25,28,29]. Unexpectedly, LEDGINs also affect late stage HIV replication [24,28,30-33], with virions produced in the presence of LEDGINs displaying severe replication defects at the level of reverse transcription (RT), nuclear import and integration [30,32,33]. Although packaging of genomic RNA and proteolytic maturation of the virus are not affected, a large proportion displays morphological defects in electron microscopy (EM) [30,32,33]. The phenotype requires the binding of LEDGINs to the LEDGF/p75 binding pocket in IN [24,30] and is mediated by enhanced multimerization of IN in the viral particles, as shown by 3 independent groups [30,32,33].

Interestingly, in a parallel approach peptides that bind LEDGF/p75 and disrupt the interaction with IN were identified by phage-display [34]. Expression of these LEDGF/p75-interacting cyclic peptides impeded HIV replication. Mechanism of action studies revealed that the peptides block integration and reduce infectivity of virus produced in cells that constitutively express the active peptide without affecting proteolytic maturation [34].

Together these observations led us to hypothesize that LEDGF/p75 may be present in the viral particle, play a role in late stage replication and hence influence LEDGIN potency. Here, we present strong evidence for a specific IN (Pol)-mediated uptake of LEDGF/p75 in HIV particles and its cleavage by HIV protease. We also demonstrate that the late stage potency of LEDGINs is not affected by the presence of LEDGF/p75. Understanding of the possible role of LEDGF/p75 or its cleavage fragments in the HIV particle awaits further experimentation.

## Results

### LEDGF/p75 is incorporated in HIV-1 particles and is a substrate of HIV-1 protease

**Figure 1:**
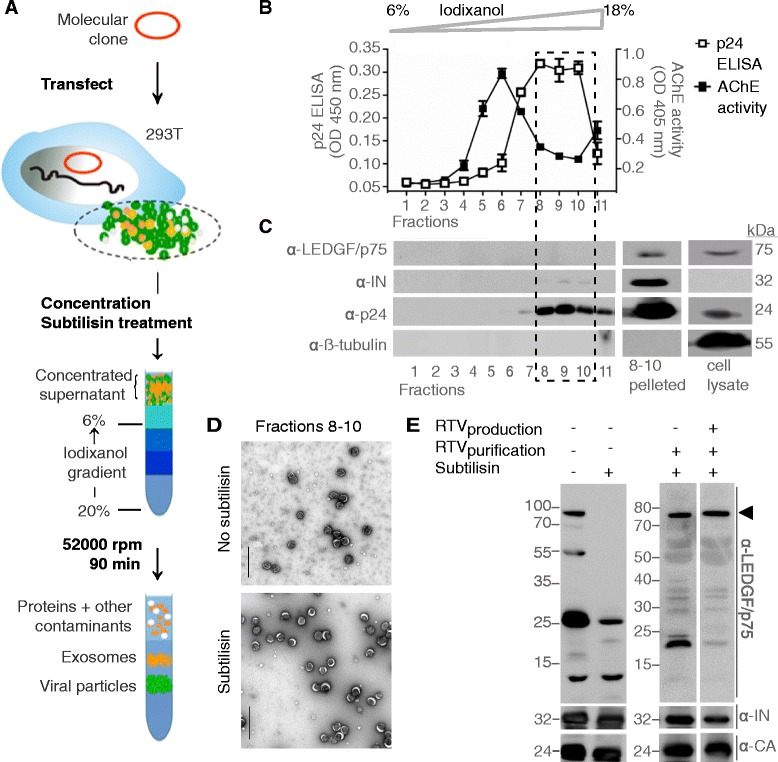
**LEDGF/p75 is incorporated in HIV-1 particles. (A)** Illustration of virus production and purification protocols. **(B)** Distribution of exosomes and HIV fractions: Exosomes as assessed by acetylcholinesterase (AChE) activity and HIV-1 as detected by p24 ELISA were effectively separated by velocity gradient purification (mean values ± standard deviations; n = 6). **(C)** Western blot analysis of individual fractions, pooled fractions 8–10 (delineated with a broken line) and producer cell lysate. **(D)** Negative staining electron microscopy (EM) of HIV fractions 8–10 purified in a Iodixanol step gradient with or without subtilisin treatment. Staining was performed with 0.5 phosphortungstic acid pH 7.2. (scale bar = 500 nm) **(E)** Western blot analysis of purified virus: Untreated virus is shown in *lane 1*, subtilisin treated virus in *lane 2*, subtilisin treated virus produced in presence of ritonavir (RTV) (added 6 hours post transfection) in *lane 3* and subtilisin treated virus with RTV added during virus production and purification in *lane 4*. Immunoblots for IN and p24 are included as control, and indicate equal loading of the samples. Full length LEDGF/p75 is indicated by an arrowhead. Lanes 1–2 were run on a 12.5% gel, lanes 3–4 on a 4-12% gel with appropriate MW markers.

We purified and concentrated HIV-1 particles by ultracentrifugation using a step gradient of 6% to 20% Iodixanol (Optiprep™) in PBS [35] (Figure 1A). Exosomes are hard to distinguish from HIV particles as for their composition and physical properties [35,36]. Our method separates viral particles from exosomes/microvesicles as shown by the distinct peaks of p24 antigen and acetylcholinesterase activity, respectively, following velocity gradient purification (Figure 1B). To evaluate LEDGF/p75 incorporation into the virions, we performed Western blot analysis on the individual fractions from the virus purification and the pooled, concentrated viral fractions (fraction 8–10) (Figure 1C). In the individual fractions IN and CA were readily detected; however, LEDGF/p75 was identified only in the pooled and additionally concentrated fraction using a specific LEDGF/p75 antibody (A300-848A, C-terminal epitope). This suggests that LEDGF/p75 is present in low abundance in the virions. Alternatively, the low avidity of the LEDGF/p75 antibody may explain the difficulty to detect the protein. Purified virus-containing fractions were additionally treated with subtilisin, a non-specific protease, to remove proteins associated with the envelope of the viral particle and to exclude LEDGF/p75 contamination. Purity of the fractions was corroborated by EM analysis (Figure 1D). However, minor contamination of these virus preparations with exosomes/microvesicles cannot be excluded.

Interestingly, apart from the 75 kDa band representing full-length LEDGF/p75 (Figure 1E, lane 1, closed arrowhead), smaller protein bands of different molecular weights were detected with the same anti-LEDGF/p75 antibody (Figure 1E, lane 1). Remarkably, following incubation of virus preparations with subtilisin (at 37°C for 18 h), only the smaller protein bands were detected with the same C-terminal antibody (Figure 1E, lane 2). This data suggest that the co-purified full-length LEDGF/p75 in the non-treated samples may have been a contaminant that is bound to the envelope of the virions and is partially digested by the non-specific protease subtilisin or that subtilisin may have non-specifically cleaved intravirion LEDGF/p75. Alternatively, prolonged exposure of LEDGF/p75 to HIV-1 protease (PR) in fully mature particles may have led to proteolytic cleavage of intravirion LEDGF/p75 by PR.

In order to address these issues and to verify our hypothesis, we performed a series of experiments. First, to verify the efficiency of removal of contaminating proteins and cellular debris by subtilisin treatment, we spiked virus preparations with recombinant Flag-LEDGF/p75 and performed subtilisin treatment. As expected, the exogenously added recombinant Flag-LEDGF/p75 was not detected by Flag antibody, indicating that subtilisin can completely degrade and remove any contaminating proteins from viral particles in our experimental system (Additional file 1: Figures S1A, S1B). Because exosomes and viral particles have comparable physical properties and are surrounded by a membrane, we had to rule out the contribution of cellular contaminants. After performing the same purification and sample processing, we only detected LEDGF/p75 fragments in viruses purified from MT-4 cells, a T-cell line, but not in the supernatant of uninfected control cells (Additional file 2: Figure S2), further indicating that LEDGF/p75 detected in the purified virion is not a contaminant. Second, in order to investigate the effect of subtilisin against intravirion proteins, we evaluated the level of viral proteins by Western blot analysis. The levels of IN and CA were similar with or without subtilisin treatment (Figure 1E), indicating that intravirion proteins are indeed protected from subtilisin degradation. Moreover, because subtilisin proteolysis is unaffected by ritonavir (RTV) treatment (Additional file 1: Figure S1A), the observed bands in Figure 1E (lane 2) are not proteolytic products of subtilisin treatment. Finally, we produced virus in the presence of 0.3 μM (6x IC_50_) RTV (PR inhibitor) and treated the purified virions with subtilisin. Full-length LEDGF/p75 was readily detected by Western blot analysis in samples treated with RTV during virus production (Figure 1E, lane 3), and was even more prominent when RTV treatment continued during purification steps too (Figure 1E, lane 4). To corroborate this, we performed Mass spectrometry analysis from RTV treated purified viruses and we were able to detect and identify LEDGF/p75 peptides (Additional file 3: Table S1, Additional file 4: Figure S3). Taken together, these observations point towards the incorporation of LEDGF/p75 in HIV particles. Furthermore, our data show that LEDGF/p75 is a substrate of HIV-1 PR irrespective of the virus producer cell lines used. Of note, only bands corresponding to IN and CA and not those of gag-products are shown to simplify the analysis. Although p24-values from virus treated with 0.3 μM RTV dropped 1 log compared to non-treated virus (as measured by 24-ELISA), we were able to detect IN and CA, since a substantial amount of virus (30 μg) was utilized for Western Blot analysis. Normalization of the virus preps was performed on protein content (BCA) and not on p24 content.

### Identification of HIV-1 protease cleavage sites in LEDGF/p75

**Figure 2:**
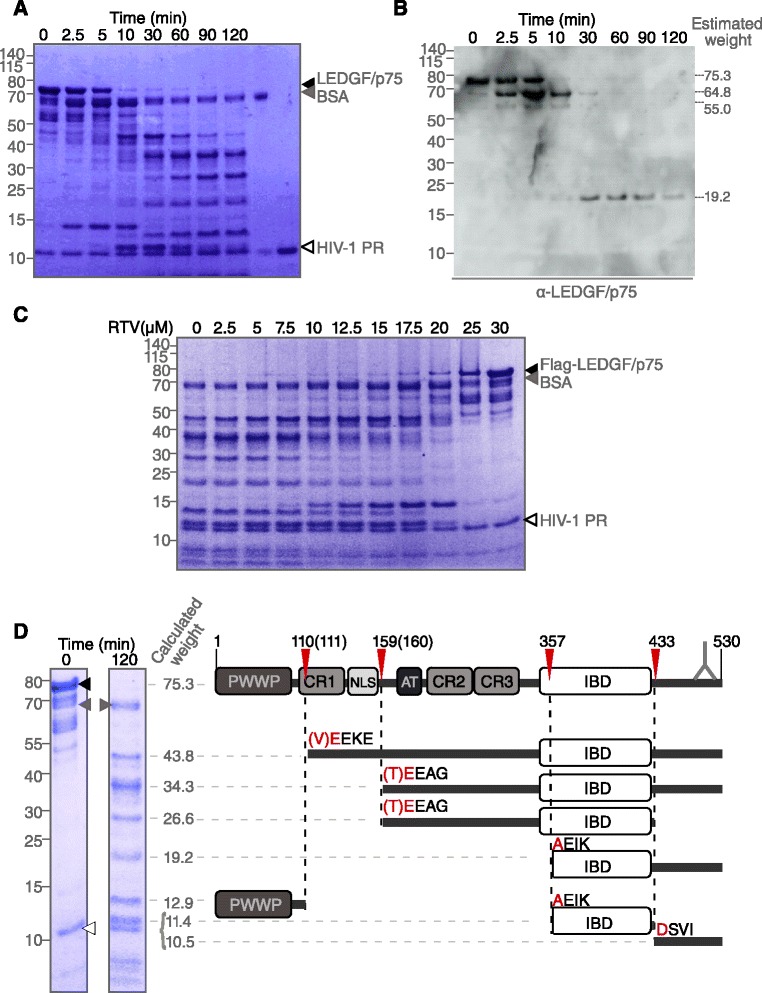
**Proteolytic cleavage sites of LEDGF/p75 by HIV-1 protease (PR).** Recombinant LEDGF/p75 proteolysis by HIV-1 PR over a period of 2 hours by **(A)** Coomassie staining after SDS-PAGE and by **(B)** immunoblotting using anti-LEDGF/p75 antibody (A300-848A). **(C)** Addition of Ritonavir (RTV) inhibits Flag-LEDGF/p75 proteolysis by HIV-1 protease in a concentration dependent manner as shown by Coomassie staining. **(D)** Schematic representation of full length LEDGF/p75, HIV-1 PR cleavage sites confirmed by N-terminal protein sequencing (red arrow-heads) and the resulting fragments. The antibody epitope is marked. Coomassie stained gel of LEDGF/p75 cleavage products with the indicated molecular mass calculated based on their relative mobility (*estimated weight*) [37]. Arrowheads indicate bands representing BSA (*grey*), LEDGF/p75 full length (*black*) and HIV PR (*open arrowheads*) in **A**, **C** and **D**.

To further characterize LEDGF/p75 as a substrate of HIV-1 PR, we performed HIV-1 PR-mediated limited proteolysis of recombinant (Flag)-LEDGF/p75 (Figure 2, Additional file 5: Figure S4). Combining Coomassie staining and Western blot analysis with anti-LEDGF/p75 (C-terminal epitope) or anti-Flag (N-terminal tag) antibody, we detected HIV-1 PR generated LEDGF/p75 fragments with apparent molecular masses of 11, 13, 14, 19, 27, 34, 44 and 65 kDa (Figures 2A, 2B, S4A, S4B). RTV protected LEDGF/p75 against proteolysis by PR in a concentration dependent manner (Figure 2C), corroborating our observation for intravirion LEDGF/p75 cleavage (Figure 1E). The RTV concentration required to inhibit LEDGF/p75 proteolysis by HIV-1 PR is higher than the concentration we normally use for its antiviral activity. In the same *in vitro* assay, we evaluated the cleavage of recombinant His-MBP-sPol_PR_D25N_ (equal mass percentage as used for Flag-LEDGF/p75) by adding active PR in *trans* with or without RTV. Once again, complete inhibition of PR is achieved only at 10 μM of RTV, suggesting that in our experimental system irrespective of the substrate nature a higher concentration of RTV is required to completely block PR (Additional file 5: Figure S4C). As an internal control we used the globular protein BSA, which is not cleaved by PR (Figure 2A,C).

Using *HIVcleave*, a HIV PR cleavage prediction program [38], we found several predicted cleavable octapeptides in LEDGF/p75 (data not shown). We then subjected highly purified recombinant LEDGF/p75 to HIV-1 PR proteolysis. We identified and sequenced 5 prominent protein bands of HIV-1 PR-derived fragments of recombinant LEDGF/p75 (Figure 2D) and mapped the HIV-1 PR cleavage sites to the predicted cleavage sites (Figure 2D, Additional file 5: Figure S4D, Additional file 3: Tables S2 and S3). In an attempt to correlate these findings with the fragments observed in lane 2 in Figure 1E, we focused on the two small fragments below 25 kDa. Taking into account that these fragments are identified in Western blot analysis using a LEDGF/p75 specific antibody that recognizes the C-terminal end (marked in Figure 2D), the two bands below the 25 kDa marker in the second lane of Figure 1E may correspond to the 10.5 and 19.2 kDa (LEDGF_357–530_) fragments. Of note, none of the HIV-1 PR cleavage sites on LEDGF/p75 overlap with the described sites for caspase-3 and −7 [39] (Additional file 3: Table S4).

### LEDGF/p75 is recruited into the viral particle through interaction with HIV integrase

**Figure 3:**
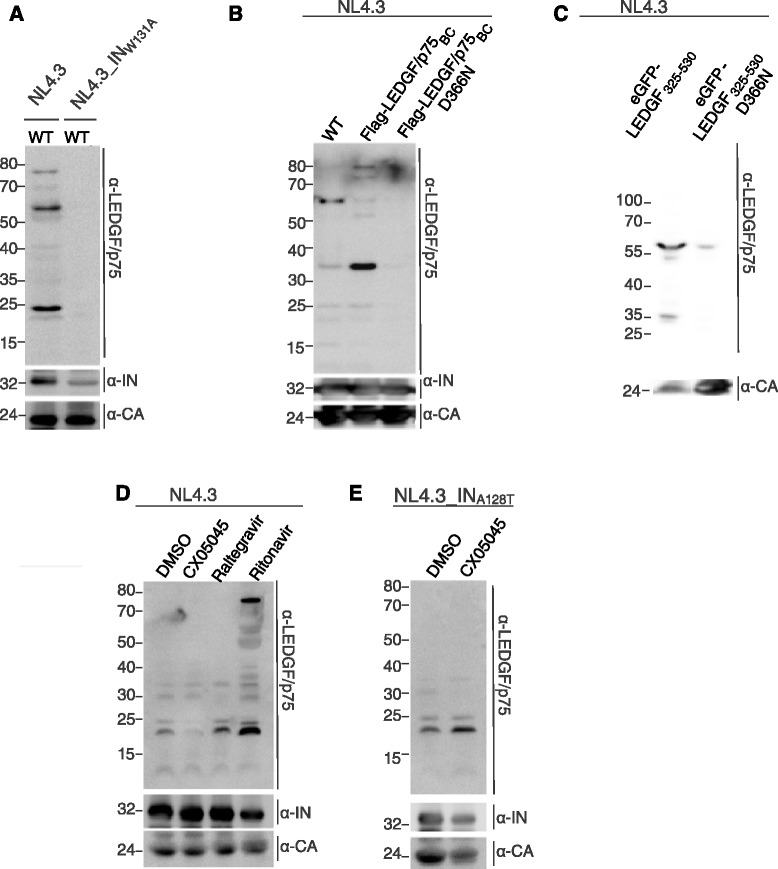
**LEDGF/p75 is recruited by integrase (Pol) into the HIV-1 particle. (A-E)** Western blot analysis of various, independently purified and subtilisin-treated virus preparations using specific antibodies to detect LEDGF/p75, IN or CA. **(A)** NL4.3 and NL4.3_IN_W131A_ produced in presence of ritonavir on 293T cells and purified. **(B)** NL4.3 produced in WT 293T cells (WT), 293T LEDGF/p75 KD cells complemented with either Flag-LEDGF/p75_BC_ or Flag-LEDGF/p75_BC_D366N. **(C)** NL4.3 produced in 293T WT cells stably expressing either eGFP-LEDGF_325–530_ or eGFP-LEDGF_325-530_D366N. **(D)** NL4.3 produced in 293T cells in the presence of DMSO, CX05045, raltegravir or ritonavir **(E)** NL4.3_IN_A128T_ produced in the presence or absence of CX05045.

To evaluate how LEDGF/p75 is recruited into the viral particle, we produced and purified a previously described NL4.3_IN_W131A_ mutant virus that carries a W131A substitution in IN disrupting the interaction between LEDGF/p75 and IN [40]. In contrast to WT virus, neither full-length LEDGF/p75 nor HIV-1 PR-derived LEDGF/p75-derived fragments were detected in NL4.3_IN_W131A_ virus produced in the presence of RTV (Figure 3A). Reciprocally, we stably back-complemented LEDGF/p75 depleted 293T cells with either Flag-tagged LEDGF/p75 (Flag-LEDGF/p75_BC_) or the D366N mutant that is unable to interact with IN (Flag-LEDGF/p75_BC_D366N). Expression was monitored by confocal microscopy, qPCR and Western blotting (Additional file 6: Figures S5A-C). We produced NL4.3, taking 293T WT cells along as control (designated as WT in Figure 3B lane 1) [41]: WT LEDGF/p75 and Flag-LEDGF/p75_BC_ were incorporated into the virion, whereas Flag-LEDGF/p75_BC_D366N was not (Figure 3B). Since LEDGF/p75 is known to interact with IN through its C-terminal part, we also evaluated virion incorporation of eGFP-LEDGF_325–530_, using WT cells expressing eGFP-LEDGF_325–530_ or eGFP-LEDGF_325-530_D366N (Figure 3C, Additional file 6: Figure S5D-F). The prominent ~60 kDa band observed corresponds to eGFP-LEDGF_325–530._ Also smaller degradation products were detected. The endogenous LEDGF/p75 fragments were detected in WT 293T controls at higher exposure (data not shown). Together, these data support recruitment of LEDGF/p75 into viral particles through direct interaction with IN.

To further verify the specificity of LEDGF/p75 incorporation in the virions, we produced virus in the presence of LEDGIN CX05045 (10 μM), raltegravir (0.06 μM) or RTV (0.3 μM) and determined LEDGF/p75 incorporation into the virions. Less LEDGF/p75 was incorporated when virus was produced in the presence of CX05045 compared with viruses produced in the presence of DMSO, raltegravir or RTV (Figure 3D). Note, that since RTV was not present, detection of full-length LEDGF/p75 was precluded (Figure 3D, lanes 1–3). Finally, we produced NL4.3_IN_A128T_ virus, a virus resistant to CX05045, in the presence or absence of the compound, and demonstrated that CX05045 did not inhibit LEDGF/p75 incorporation into NL4.3_IN_A128T_ particles (Figure 3E), further indicating that LEDGF/p75 recruitment into HIV-1 particles is specific and depends on interaction with IN.

Finally, we estimated the ratio of LEDGF/p75 and eGFP-LEDGF_325–530_ to IN in the virions (Additional file 7: Figures S6A, S6B). The stoichiometry of LEDGF/p75 to IN and eGFP-LEDGF_325–530_ to IN were approximately 1:250 and 1:140, respectively. However, it is important to take into consideration that the effect of PR-mediated degradation of full-length LEDGF/p75 confounds this analysis leading to underestimation of the protein. Nevertheless, these results suggest that LEDGF/p75 is specifically recruited by IN and that abrogation of the LEDGF/p75-IN interaction in virus producer cells interferes with the uptake of LEDGF/p75 in the virion.

### LEDGF/p75 interacts with the HIV-Pol polyprotein

**Figure 4:**
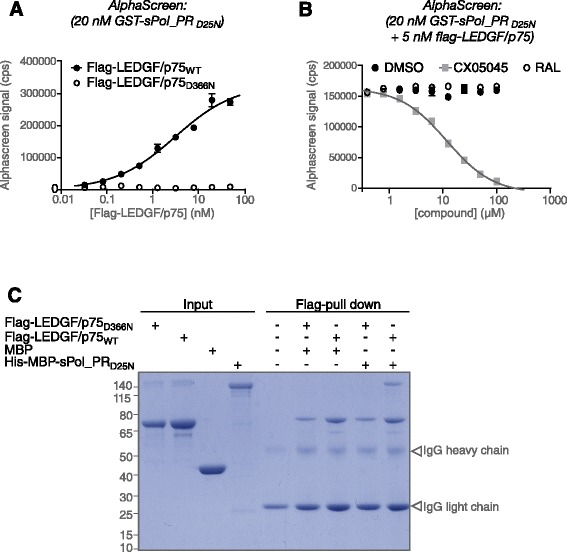
**Interaction between LEDGF/p75 and HIV Pol.** Direct interaction of LEDGF/p75 with HIV-1 Pol polyprotein as determined by an AlphaScreen PPI assay. **(A)** Titration of Flag-LEDGF/p75_WT_ or Flag-LEDGF/p75_D366N_ against 20 nM GST-sPol_PR_D25N_. LEDGF/p75_WT_ binds Pol with an apparent K_D_ of 3.1 ± 1.1 nM, whereas the D366N point-mutation abolishes interaction. **(B)** Titration of CX05045, raltegravir (RAL) and DMSO against a constant background of 5 nM Flag-LEDGF/p75_WT_ and 20 nM GST-Pol_PR_D25N_. CX05045 inhibit the interaction with an IC_50_ of 10.7 μM (95% CI 9.5-12.0). Data represent mean values ± standard deviations of duplicate measurements in a representative experiment. **(C)** Pull-down of His-MBP-sPol_PR_D25N_ using recombinant Flag-LEDGF/p75_WT_ or Flag-LEDGF/p75_D366N_.

We demonstrated that recruitment of LEDGF/p75 into HIV virions requires interaction with IN. Moreover, it is known that LEDGF/p75 interacts with the catalytic core of IN [41]. This would imply that IN dimers are pre-formed at the precursor (Gag)-Pol polyprotein level in virus producing cells. In fact, we and others have recently shown that LEDGINs bind HIV-1 Pol polyprotein and enhance IN/Pol multimerization [30,32,33]. Moreover, in a recent global virus-host interaction proteomics analysis, LEDGF/p75 was identified as one of the host proteins co-immunoprecipitated with HIV-1 Pol polyprotein [21]. We analyzed the interaction between HIV-1 Pol polyprotein and LEDGF/p75 by AlphaScreen using recombinant Glutathione S-Transferase tagged (GST)-Pol polyprotein with a catalytically dead PR_D25N_ (GST-sPol_PR_D25N_) and Flag-LEDGF/p75. We observed direct and specific binding between LEDGF/p75 and Pol with an apparent *K*_d_ of 3.1 ± 1.1 nM and a Hill coefficient of 0.65 ± 0.08 (Figure 4A), indicating negative cooperativity. Considering that the LEDGF/p75 binding requires an IN dimer interface, these results suggest the existence of one high and one low affinity LEDGF/p75 binding site on a Pol dimer. In contrast, LEDGF/p75_D366N_ failed to interact (Figure 4A), corroborating specificity of the assay and the findings in the virion (Figure 3B). Next, we evaluated the effect of CX05045, raltegravir or DMSO on the LEDGF/p75-Pol interaction (Figure 4B). CX05045 inhibited the LEDGF/p75-Pol interaction in a concentration dependent manner with an IC_50_ of 10.7 μM (95% CI 9.5-12.0) and a Hill coefficient of 1.0. Neither DMSO nor raltegravir affected the interaction. In pull-down assays we also observed that Flag-LEDGF/p75, but not Flag-LEDGF/p75_D366N_ interacts with His-MBP-tagged synthetic Pol polyprotein (Figure 4C: His-MBP-sPol_PR_D25N_).

Overall, on the basis of the direct interaction between HIV-1 Pol polyprotein and LEDGF/p75 *in vitro*, along with the requirement of IN (Pol) interaction for virion incorporation of LEDGF/p75 in the virus producer cells, we conclude that LEDGF/p75 can be specifically incorporated into the virions through an interaction with either IN or its precursor Pol protein.

### LEDGIN potency is independent of the presence of LEDGF/p75 in virus producer cells

**Figure 5:**
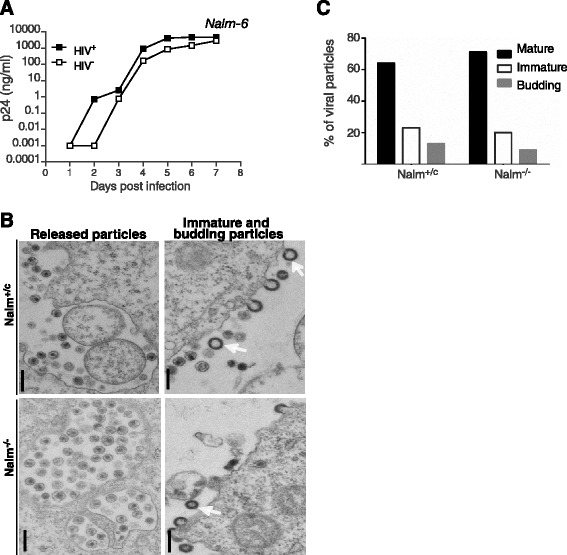
**Effect of LEDGF/p75 on replication capacity of newly produced HIV-1 virions. (A)** Replication of HIV^+^ (virus produced in WT Nalm 6 cells) and HIV^−^ (virus produced on Nalm^−/−^ cells), after normalization for p24, was monitored in Nalm^+/c^ cells by p24 ELISA. **(B)** Thin section TEM images of HIV-1 particles generated by NL4.3 infected Nalm^+/c^ or Nalm^−/−^ cells. Arrows indicate immature particles (scale bar = 200 nm). **(C)** Viral particles were classified into mature particles with conical capsid (*black bars*), immature particles (*white bars*), and budding particles (*gray bars*), and the percentage of each category was calculated relative to the total number of particles (HIV^−^; 150, HIV^+^; 350 viral structures per condition) analyzed.

As LEDGF/p75 incorporation into viral particles was hampered by LEDGIN CX05045, we investigated if LEDGIN potency at the late stage is dependent on the presence of LEDGF/p75 fragments in viral particles. Therefore we infected TZM-bl cells, containing a HIV-1 LTR driven luciferase reporter gene, with NL4.3 produced in LEDGF/p75_KD_ or WT 293T cells with or without 0–13.5 μM CX05045. CX05045 reduced virus infectivity irrespective of the presence of LEDGF/p75 in virus producing cells (Table 1), excluding a major contribution of LEDGF/p75 to the potency of LEDGINs on late stage HIV replication. These data are in agreement with previous reports [26,32,33,42]. Furthermore, the absence of LEDGF/p75 in virus producer cells did not result in major defects in virus infectivity (Figure 5A) or morphological impairments (Figure 5B, 5C). Likewise, HIV^−^, virus produced on LEDGF/p75 knock out cells (Nalm^−/−^), showed only modest defects in replication capacity compared to HIV^+^, virus produced on WT cells (Nalm^+/c^).

**Table 1:** **The potency of LEDGINs is not affected by the presence of LEDGF/p75 HIV producer cells**

**Producer cell line**	**EC**_**50**_ **CX05045 (μM)** ^**1**^
WT	0.6 (95% CI: 0.5-0.8)
LEDGF/p75_KD_	0.8 (95% CI: 0.6-1)

^1^Effective concentration in μM required to reduce Tat-driven firefly luciferase activity by 50% in TZM-bl cells at 40 hpi. The table shows means and the 95% confidence interval (95%CI).

Together these data suggest that the recruitment of LEDGF/p75 into viral particles seems not an essential process for generation of infectious particles or late stage LEDGIN potency.

## Discussion

The interaction of LEDGF/p75 with HIV-1 Pol [21], the observations on the late stage effect of LEDGINs [24,30-33], and our data on cyclic peptides binding to LEDGF/p75 [34], prompted us to investigate if LEDGF/p75 is incorporated in viral particles. Our study revealed that LEDGF/p75 is indeed incorporated into HIV virions in an IN-dependent manner and that LEDGF/p75 is an authentic substrate of HIV-1 PR. We provide evidence that LEDGF/p75 already interacts with IN when part of the Pol polyprotein, probably driving the specific incorporation of LEDGF/p75 in HIV virions. Intravirion LEDGF/p75 did not alter the apparent potency of LEDGINs during late stage infection. No drastic impact of intravirion LEDGF/p75 on HIV infectivity in cell lines could be demonstrated so far.

In a series of experiments, we revealed that LEDGF/p75 can be detected in highly purified HIV virions (Figure 1), and is a substrate for HIV PR, as we detected several HIV-1 PR-derived LEDGF/p75 fragments in purified virus preparations. Addition of the PR inhibitor, RTV, allowed detection of full-length LEDGF/p75 in these virus particle preparations (Figures 1 and 2). We identified HIV PR cleavage sites for the prominent fragments using N-terminal sequencing (Figure 2, Additional file 5: Figure S4, Additional file 3: Table S3), even though multiple other sites can be identified by *HIVcleave* prediction. LEDGF/p75 fragments were absent or diminished in viruses containing IN_W131A_, an IN mutant incapable of binding to LEDGF/p75, or in viruses produced from cells expressing mutant LEDGF/p75_BC_D366N, defective for interaction with HIV-1 IN (Figure 3). Furthermore, as PR is unlikely to be active prior to assembly to generate mature Gag and Pol products, the direct interaction between LEDGF/p75 and HIV-1 Pol (Figure 4) and the identification of LEDGF/p75 in immature virion preparations produced in presence of RTV are compatible with the requirement of a LEDGF/p75-Pol interaction for virion incorporation. We propose a model whereby LEDGF/p75 is incorporated in HIV virions through an interaction with dimeric IN core domain, already present in a Pol polyprotein dimer. Although LEDGF/p75 is a nuclear protein, it is synthesized in the cytoplasm and other examples of nuclear proteins that are detected in purified HIV particles exist. Such proteins include INI-1 [43], Ku70 [44], Ku80 and U5 small nuclear ribonucleoprotein [45]. Moreover, although detection of LEDGF/p75 in the supernatant of cell cultures (Figure 1) may result from cell damage, LEDGF/p75 and related HRP-proteins have been shown to be secreted [46,47].

Although more than 300 human proteins have been identified in HIV viral particles (summarized in [48], reviewed in [36]), LEDGF/p75 has not been detected [36,45,48,49]. HIV protease-mediated cleavage of LEDGF/p75 might be responsible for this lack of detection. Poor avidity of the available LEDGF/p75 antibodies hampers the detection in immunoblots requiring the use of large amounts of concentrated virions. Furthermore, the low abundance of LEDGF/p75 in viral particles (Additional file 7: Figure S6) prompted us to use a specific approach for the MS analysis, focusing on part of the SDS-page gel corresponding to 75 kDa proteins for MS-analysis.

Nevertheless, taking into account the number of Pol molecules per virion, we estimate that only 1 to 2.5 molecules of LEDGF/p75 are present per viral particle. It is estimated that there are ~5,000 copies of Gag molecules per immature virion with a Gag-Pol to Gag ratio of 1:10 to 1:20. Considering 100% efficiency of proteolytic maturation of the precursor Gag-Pol and Pol polyproteins, there would be a maximum of 250 to 500 copies of RT and IN [50]. We estimate the LEDGF/p75 to IN in the range of 1:250, estimated by semiquantitative immunoblot analysis, although PR cleavage may result in an underestimation of the absolute amount of LEDGF/p75. In any case, even though the detected intravirion LEDGF/p75 appears specifically incorporated and not the result of a contamination, only few LEDGF/p75 molecules would be present per viral particle.

Although we show that LEDGINs hamper LEDGF/p75 incorporation in the viral particle (Figure 3D), NL4.3 virus produced in LEDGF/p75_KD_ cell lines showed a similar sensitivity to LEDGINs compared to virus produced on WT cells (Table 1). Similar results were obtained by Balakrishnan *et al.* [33], Jurado *et al.* [32], Fadel *et al.* [42] and Sharma *et al.* [26]. LEDGINs do enhance IN multimerization in the absence of LEDGF/p75. Since a relatively high concentration of CX05045 (10-fold higher than for IN) seems to be required to displace LEDGF/p75 from Pol polyprotein (Figure 3C), a more subtle contribution of virion incorporated LEDGF/p75 to the mechanism of action of LEDGINs cannot be excluded.

LEDGF/p75 depletion in HIV-producer cells did not lead to aberrant morphological changes in virus particles as detected by TEM (Figure 5B,C) and no major reduction in viral infectivity (Figure 5A), as reported before [42].

## Conclusion

In conclusion, we show that LEDGF/p75 is specifically recruited by IN/Pol into HIV-1 particles and that LEDGF/p75 is a substrate for HIV-1 protease. So far, no critical role can be attributed to LEDGF/p75 in the generation of infectious viral particles. Intravirion LEDGF/p75 has apparently no major effect on late stage potency of LEDGINs.

## Methods

### Reagents

Antiviral compounds: LEDGINs (synthesized by Centre for Drug Design and Development (CD3), KULeuven R&D, Leuven, Belgium), AZT, raltegravir and RTV (obtained from the AIDS Research and Reference Reagent Program, Division of AIDS, NIH).

Chemicals: subtilisin, acetylthiocholine iodide substrate and 5, 5′-dithiobis-2-nitrobenzoic acid (Ellman’s reagent, DTNB) (purchased from Sigma-Aldrich, Benelux B.V., Belgium).

Antibodies: Anti-LEDGF/p75 (rabbit, A300-848A, Bethyl laboratories, Montgomery, TX), anti-β-tubulin (mouse, T-4026, Sigma-Aldrich, St Louis, MO), anti-HIV-1 CA^p24^ (mouse, #24-2, AIDS Research and Reference Reagent Program, Division of AIDS, NIAID, NIH), anti-HIV-1 IN (mouse, IN-2 (ab66645), Abcam plc, Cambridge Science Park, Cambridge, UK or monoclonal antibody (8G4) from Division of AIDS, NIAID, NIH contributed by Dr. Dag E. Helland) and anti-Flag (mouse, M2 monoclonal antibody (F3165) Sigma-Aldrich, St. Louis, MO, USA) were used.

### Cell culture and generation of cell lines

293T, and HeLa TZM-bl (obtained from the AIDS Research and Reference Reagent Program, Division of AIDS, NIH: TZM-bl from Dr. John C. Kappes, Dr. Xiaoyun Wu and Tranzyme Inc.) cells were maintained in Dulbecco’s modified Eagle medium (GIBCO BRL, Merelbeke, Belgium) supplemented with 8% fetal calf serum (FCS; Sigma-Aldrich, Bornem, Belgium) and 50 μg/ml gentamicin (GIBCO BRL). MT-4 and Nalm-6 cells were grown in RPMI 1640 (GIBCO BRL) supplemented with 12% FCS and 50 μg/ml gentamicin. All cell lines were grown in a humidified atmosphere with 5% CO_2_ at 37°C.

A SIV-based lentiviral vector (pGAE SFFV-GFP-IRES-tCD34-2xshL3mir) was used to create stable LEDGF/p75_KD_ in 293T cells. pGAE_CAG-eGFP-WPRE [51] (kind gift from Didier Nègre, Ecole Normale Supérieure, Lyon) was used as backbone and digested with *Esp3*I and *BamH*I to replace the CMV early enhancer/chicken β-actin (CAG) promoter by a multiple cloning site (MCS) containing BsiWI, Eco47III and BamHI restriction sites to result in pGAE_MCS-eGFP-WPRE, which served as a backbone for further cloning. Next, eGFP was replaced by SFFV-ZeoR-IRES-tCD34-2xshL3mir, which was digested from pCH_SFFV-ZeoR-IRES-tCD34-2xshL3mir [52] using AgeI and ClaI and ligated into the backbone which was digested using the same restriction enzymes resulting in pGAE SFFV-ZeoR-IRES-tCD34-2xshL3mir. The SIV-based lentiviral vector was produced by transfecting 15 μg transfer plasmid, 15 μg packaging plasmid and 5 μg VSV.G envelop plasmid per culture dish carried out as described before [53]. 293T cells were transduced at high MOI using pGAE SFFV-ZeoR-IRES-tCD34-2xshL3mir to create stable LEDGF/p75_KD_ 293T cells. To generate 3xFlag-LEDGF/p75_BC_ and 3xFlag-LEDGF/p75_BC_D366N cell lines, we transduced the LEDGF/p75_KD_ cells with pCMHWS-3xFlag-LEDGF/p75-IRES-HygroR and pCMHWS-3xFlag-LEDGF/p75-IRES-HygroR lentiviral vectors, respectively. LEDGF/p75 expression was confirmed by immunocytochemistry, Western blot and RT-qPCR.

### Virus production from LEDGF/p75 KO cells

To generate HIV^+^ and HIV^−^, 1x10^6^ Nalm^+/c^ and Nalm^−/−^ cells (as described by Schrijvers et al. [17]) were challenged with HIV-1_NL4–3_ at high MOI, washed twice with PBS after two days and resuspended in culture medium; at day 7–9 the supernatant was filtered through a 0.45 μm filter (Millipore) and harvested. Three independent HIV^+^ and HIV^−^ productions tested pairwise and gave reproducible results. Genotypically both viruses have WT IN as verified by sequencing.

### Virus production by transfection

Production of the HIV-1 molecular clone NL4.3 was carried out by transfecting 293T cells as described before [30,53]. Briefly, 5.5 × 10^6^ cells were plated and transfected with 20 μg of plasmid per cell culture dish in OptiMEM. The transfection mixture was added directly on the cells drop by drop. 6 h post transfection, the transfection medium was replaced with OptiMEM supplemented with 50 μg/ml gentamicin. 72 h post transfection, cell-free supernatant was harvested, filtered through 0.22 μm filters, and used directly or stored at −80°C until use.

### Infectivity assays

To determine infectivity, Nalm^+/c^ cells were infected with equal amounts of viruses normalized for p24 antigen and washed 3 times 6 hours post infection. Virus replication was monitored by quantifying p24 level in the supernatant on successive days using p24 ELISA (Innogenetics, Ghent, Belgium).

To determine the EC_50_ in TZM-bl cells, 2x10^4^ cells were infected with equivalent amounts of virions produced in presence of a CX05045 1:3 dilution series. Cells were lysed in buffer containing 50 mM Tris/HCl, pH 7.3, 200 mM NaCl, 0.2% NP40 and 5% glycerol and analyzed for firefly luciferase activity (ONE-Glo™, Promega, Belgium) according to the manufacturer’s instructions. Readouts were normalized for protein content as determined by a BCA-assay (BCA Protein Assay Kit, Thermo Scientific).

### Purification of HIV particles

Twenty to twenty-five 10-cm dishes of 80% confluent 293T cells were transfected with NL4.3, NL4.3_IN_A128T_ or NL4.3_IN_W131A_ as described above. For some of the experiments, RTV (300 nM) was added 6 h post transfection and added during the purification and subtilisin treatment step to prevent LEDGF/p75 cleavage by PR. At 72 h post transfection, supernatants were harvested and filtered through 0.22 μm filters (Millipore). Viruses were pelleted by ultracentrifugation (31,000 rpm, 45 minutes, Ti70 rotor, Beckman Coulter), resuspended and treated with 500 μl subtilisin (1 mg/ml) (Sigma, Belgium) in 20 mM Tris–HCl (pH 8.0), 150 mM NaCl and 1 mM CaCl_2_ for 18 hours at 37°C to remove any contaminating sticky proteins. Subtilisin was inactivated by adding 500 μl of DMEM supplemented with 10% FCS, 20 mM EDTA and 5 μg/ml of PMSF at room temperature for15-30 minutes. The subtilisin treated virus was then applied to an iodixanol (Optiprep™) step gradient (6–20.4%) and ultracentrifuged using the Ti70 rotor (52,000 rpm, 90 minutes). Fractions were taken from top and one-tenth of the fractions were immediately used to perform the Ellman acetylcholinesterase assay [54], while one-twentieth of the fractions were used for p24 ELISA to distinguish the fractions containing exosomes and pure viral particles, respectively. Fractions 8–10 were pooled, pelleted by ultracentrifugation (31,000 rpm for 45 minutes) and lysed with 1% SDS. Typical protein yields of 2–5 mg/ml were obtained and the samples were subjected to SDS-PAGE and Western blot analysis. Production of virus in MT-4 cells was achieved through infection with WT NL4.3 virus. For the purification, the same protocol was followed as for purification of virus produced in 293T cells.

### Ellman acetylcholine esterase activity assay

To evaluate the virus purification procedure and in particular the separation of exosomes from virus particles, we optimized a previously described assay [35,54]. Acetylcholinesterase (AChE) hydrolyzes acetylthiocholine into thiocholine and acetate. The assay measures the activity of AChE present in intact microvesicles by quantifying the absorbance at 405 nm. 100 μl of the fractions were transferred to 96-well plates and incubated at 37°C for 10 minutes. Then 100 μl of a mixture of 100 mM sodium phosphate buffer, pH 8.0, 75 mM acetylthiocholine iodide substrate (Sigma) and 10 mM 5, 5′-dithiobis-2-nitrobenzoic acid (Ellman’s reagent, DTNB, Sigma) in a ratio of 150:2:5 was added to each well and incubated for an extra 10 minutes at 37°C. The plates were read using the Perkin Elmer Envision platform at 405 nm.

### Semi-quantitative p24 ELISA

To define HIV-1 particle containing fractions and control purification efficiency, we performed a semi-quantitative p24 ELISA on the fractions using the Alliance HIV-1 p24 ELISA kit (Perkin Elmer). For this assay, we boiled 50 μl of each fraction at 95°C for 5 minutes prior to transfer to immunosorbent 96-well plates (NUNC, Denmark). After sealing the plates were incubated overnight at 4°C. Next, we removed the supernatant and blocked non-specific signal with 5% donkey serum for 30 minutes at room temperature. The subsequent experimental procedures and plate reading were performed following the manufacturer’s instructions.

### *In vitro* HIV-1 protease (PR) assay and detection of the cleavage products

Affinity purified recombinant HIV-1 PR was a kind gift from Dr. P. Řezáčová, Institute of Organic Chemistry and Biochemistry, Academy of Sciences of the Czech Republic. 20 μg of purified recombinant (Flag)-LEDGF/p75 protein was incubated with 2 μg HIV-1 PR in 50 μl reaction buffer (50 mM sodium acetate buffer; pH 4.9, 200 mM NaCl and 0.002% BSA) for 2 h at 37°C. The specificity of proteolysis was controlled by incubating HIV PR with different concentrations of RTV (2.5-50 μM) for 30 minutes before adding LEDGF/p75. To monitor the order and kinetics of LEDGF/p75 proteolytic cleavage, we followed proteolysis over time and analyzed the protein bands. In all cases, PR was immediately inactivated by adding Laemmli loading buffer and heating at 98°C for 3–5 minutes and the mixtures were separated using 4-12% SDS-PAGE. Bands were visualized either by Coomassie staining or by Western blotting using anti-LEDGF/p75 (A300-848A, Bethyl Laboratories. Inc, recognizing aa 480–530) or anti-Flag antibody (detects fragments with intact N-terminus). N-terminal sequence analysis of the cleaved LEDGF/p75 derivatives was performed after blotting on PVDF membrane and staining with Coomassie brilliant blue. Individual bands were cut from the membrane, destained in methanol and the amino acid sequence was determined after elution of each band and subjected to by automated Edman degradation using a capillary 491cLC protein sequencer (Applied Biosystems).

To evaluate the effect of subtilisin on purified LEDGF/p75, 20 μg Flag-LEDGF/p75 was incubated 0, 2 or 18 hours with 13.33 ng (1:1500 enzyme to substrate ratio) or 25 μg (1:0.8 enzyme to substrate ratio) of subtilisin in 50 μl reaction buffer (20 mM Tris–HCl pH 8.0, 150 mM NaCl and 1 mM CaCl_2_). Inactivation and visualization was performed as described for the PR assay. To evaluate the effect of subtilisin on LEDGF/p75 absorbed to the virion surface, NL4.3 virus was pelleted, spiked during 4 hours with or without 40 μg Flag-LEDGF per 100 μL of virus and treated with or without subtilisin (1mg/mL) in subtilisin reaction buffer during 18 hours. Western blotting was performed as described above.

### Purification of recombinant proteins

GST-sPol_PR_D25N_, His-MBP-sPol_PR_D25N,_ Flag-LEDGF/p75 WT and Flag-LEDGF/p75_D366N_ were purified as described before [9,30]. MBP was purchased from New England Biolabs.

### AlphaScreen protein-protein interaction assay

AlphaScreen (PerkinElmer) is a bead-based technology that allows studying biomolecular interactions. Briefly, all proteins, compound controls and beads were diluted to their respective working stocks in assay buffer (25 mM Tris/HCl pH 7.5, 150 mM NaCl, 1 mM dithiothreitol, 1 mM MgCl_2_ 0.1% (w/V) BSA, 0.1% (V/V) Tween 20). 5 μL buffer/compound, 5 μL GST-Pol_PR_D25N_ and 5 μL Flag-LEDGF/p75 were added to wells of a 384-well OptiPlate (PerkinElmer). The plate was sealed and left to incubate for 1 h at 4°C. Next, 10 μL of a mix of glutathione donor and anti-Flag acceptor AlphaScreen beads was added (20 μg/mL final each), the plate was resealed and incubated for 1 more hour at 23°C. Alpha signal was read out on an EnVision Multilabel plate reader (PerkinElmer) and data were analyzed using Prism 5.0 (GraphPad).

For the titration experiments, GST-sPol_PR_D25N_ was used at a constant concentration of 20 nM while WT and D366N Flag-LEDGF/p75 were titrated from 50 nM downward in a 1:2.5 dilution series. In the compound competition assays, both GST-Pol_PR_D25N_ and WT Flag-LEDGF/p75 were kept constant at 20 and 5 nM respectively and CX05045, raltegravir or DMSO were titrated in a 1:2 dilution series starting at 100 μM.

### Pull-down assays

5 μg Flag-tagged LEDGF/p75_WT_ and LEDGF/p75_D366N_ were first bound to anti-Flag M2 Affinity Gel (Sigma) and incubated 10 minutes on ice. The slurry was then incubated for 4 hours with 10μg of His-MBP- sPol_PR_D25N_ or MBP in 250 μL binding buffer (25 mM Tris pH 7.4, 150 mM NaCl, 0.1% NP40, 1 mM MgCl_2_, 2 mM DTT).

After washing 3 times with wash buffer (25 mM Tris pH 7.4, 300 mM NaCl, 0.1% NP40, 1 mM MgCl_2_, 2 mM DTT), the proteins bound to the beads were eluted with elution buffer (binding buffer with 1% SDS) and were subjected to SDS-PAGE analysis and visualized by Coomassie staining.

### Gel electrophoresis and immunoblot analysis for LEDGF/p75 uptake in virions

Protein samples were prepared in 1% SDS. Next, 30 μg of total protein content was loaded in each sample lane and proteins were separated by SDS-PAGE (4-12% bis-tris or 12.5% tris-glycine). For LEDGF/p75 and HIV-1 IN quantification a dilution series of recombinant Flag-tagged LEDGF/p75 or His-IN was loaded next to respectively 30 μg of viral lysate for LEDGF/p75 detection, 15 μg for eGFP-LEDGF_325–530_ detection or 7.5 μg for IN detection, purified and concentrated as described above. Protein markers were PageRuler Prestained Protein Ladder and PageRuler Prestained Protein Ladder from ThermoScientific. Proteins were detected with the respective antibody: rabbit anti-LEDGF/p75 (1:100 for viral lysate and 1:1000 for cell lysate, Bethyl Laboratories. Inc), mouse monoclonal anti-HIV-1 IN (IN2, 1:10,000 for viral lysates and 1:2000 for cell lysates, Abcam), mouse anti-HIV-1 CA (1:10,000, AIDS reagents Program). Visualization was performed using chemiluminescence (Pierce ECL, Thermo Scientific).

### Electron microscopy

Negative staining EM was performed with 50 μl of inactivated virus fractions applying the grid on drop method (Laue and Bannert 2010). Four hundred mesh copper grids (Plano, Wetzlar, Germany) filmed with pioloform were coated with carbon and were glow-discharged to increase hydrophilicity and particle adherence before incubation with the virus suspension (for 10 minutes). After washing in 5 droplets of water, grids were stained with 1% phosphortungstic acid, pH 7.2 and analyzed with a TEM (Tecnai 12 BioTwin, FEI). For thin section EM. 1×10^6^ Nalm-6 cells were infected with the NL4.3 for 2 days and then washed with PBS and incubated with fresh medium. 30 hours post washing, cells were pelleted and fixed overnight at 4°C with 2.5% glutaraldehyde. Cell pellets were post-fixed with OsO4 (1% in ddH2O; Plano, Wetzlar, Germany), block-stained with uranyl acetate (2% in ddH2O; Merck, Darmstadt, Germany), dehydrated stepwise in graded alcohol, immersed in propylenoxide and embedded in Epon (Serva, Heidelberg) with polymerisation at 60 °C for 48 h. Ultrathin sections (60–80 nm) were cut using an ultramicrotome (Ultracut S or UCT; Leica, Germany) and stained with 2% uranyl acetate and lead citrate. Transmission electron microscopy was performed with an EM 902 (Zeiss) operated at 80 kV and the images were digitised using a slow-scan charge-coupled-device camera (Pro Scan; Scheuring, Germany).

## Additional files

Additional file 1: Figure S1. Effect of subtilisin on LEDGF/p75 proteolysis. (A) Coomassie stained SDS-PAGE gel showing that LEDGF/p75 degradation by subtilisin is not inhibited by ritonavir (RTV). Lane 1-3: Limited proteolysis of LEDGF/p75 (20 μg) was performed at 37°C for different time intervals (0-2-18 hours) with 13.33 ng of subtilisin (1:1500 (w/w) ratio of enzyme to substrate). Lane 4: the proteolysis was performed for 18 hrs using 1 mg/ml subtilisin (enzyme to substrate ratio 1:0.8); this concentration was used during virus purification. Bands representing BSA and subtilisin are indicated with arrowheads. (B) Western blot analysis with anti-Flag antibody of subtilisin treated virus preparations spiked with recombinant Flag-LEDGF/p75. NL4.3 was spiked 4 hours with buffer (lane 1) or Flag-LEDGF/p75 (lane 2, 3) at 4°C and subsequently incubated at 37°C for 18 hours with (lane 3) or without (lane 1, 2) subtilisin. Additional file 2: Figure S2. Detection of LEDGF/p75 in purified virions produced in a T-cell line. Supernatant of HIV-1 IIIB (lane 1) and mock infected (lane 2) MT-4 cells was harvested, treated with subtilisin and purified over an iodixanol velocity gradient. (A) Acetylcholinesterase (AchE) activity and p24 content of the different fractions show an effective separation of exosomes and HIV virions. (B) Fractions 8-10 were pooled and subjected to Western blot analysis using anti-LEDGF/p75, anti-IN or anti-CA antibody. Additional file 3 Table S1. LEDGF/p75 peptides detected from purified HIV virions using Mass Spectrometry. **Table S2.** Determination of the molecular mass of LEDGF/p75 fragments generated by HIV-1 PR *in vitro.* **Table S3.** N-terminal sequencing of LEDGF/p75-derived fragments following HIV-1 PR digestion. **Table S4.** Identified cleavage site sequences and cleavage prediction algorithm of octapeptides derived from LEDGF/p75 cleavage by HIV-1 PR. Additional file 4: Figure S3. LEDGF/p75 detected in purified HIV virions using Mass Spectrometry. Peptides described in Additional file 3: Table S1 mapped to LEDGF/p75. Confirmed (*red*) and predicted (*orange*) protease cleavage sites are indicated, as well as the integrase interacting D366 amino acid (*blue*). Protease cleavage sites were predicted with *HIVcleave* [38,55]. Additional file 5: Figure S4. In vitro limited proteolysis of LEDGF/p75 by HIV-1 PR and determination of molecular mass of cleavage products generated by HIV-1 PR. (A, B) HIV-1 PR cleavage products of pure recombinant Flag-LEDGF/p75 separated by SDS-PAGE. Coomassie brilliant blue staining (A) revealed the same cleavage pattern as untagged LEDGF/p75, while Western blot analysis (B) using anti-Flag antibody recognized N-terminal fragments or C-terminal fragments using anti-LEDGF/p75 antibody. Bands representing BSA and HIV-1 PR are indicated with arrowheads. (C) Cleavage of recombinant His-MBP-sPol_PR_D25N_ by recombinant HIV-PR is inhibited by high concentrations of ritonavir (RTV). (D) Standard curve generated from the reference protein marker bands (R2 = 0.9982) as described before [1]. Molecular mass (kDa) of the different fragments of LEDGF/p75 and reference full-length proteins are indicated on the standard curve. Additional file 6: Figure S5. Characterization of the expression of different LEDGF/p75 constructs. (A, B, C) LEDGF/p75-depleted cells (LEDGF/p75KD) were back-complemented with lentiviral vectors encoding either Flag-LEDGF/p75 (LEDGF/p75_BC_) or Flag-LEDGF/p75_BC_D366N. (A) Fluorescence microscopy images of cells stained with anti-LEDGF/75 antibody (*red*). Nuclei were stained with DAPI (*blue*). The levels of protein and mRNA for the different LEDGF/p75 constructs were determined by RT-qPCR (B) and Western blot analysis (C), respectively. β-tubulin was included as a loading control. (D, E, F) Stable overexpression of eGFP-LEDGF_325-530_ or eGFP-LEDGF_325-530_D366N in 293T cells verified with (D) fluorescence microscopy (anti-LEDGF/p75 antibody: *red*; eGFP: *green*; DAPI: *blue*), (E) RT-qPCR or (F) Western blotting (LEDGF/p75: *black arrowhead*, eGFP-LEDGF_325-530_: *open arrowhead*). Additional file 7: Figure S6. Quantification of LEDGF/p75 or eGFP-LEDGF_325-530_ in HIV particles. (A, B) Western blot of recombinant Flag-LEDGF/p75 (upper lanes) or His-IN (lower lanes) in a 1:3 dilution series, next to HIV virions harvested, subtilisin treated, purified and concentrated from WT 293T cells (A) or cells overexpressing eGFP-LEDGF_325-530_ or eGFP-LEDGF_325-530_D366N (B) using anti-LEDGF/p75 (upper lanes) or anti-IN (lower lanes) antibody.
