# Correction to: HIV-1 IN/Pol recruits LEDGF/p75 into viral particles

**DOI:** 10.1186/s12977-020-00531-3

**Published:** 2020-07-29

**Authors:** Belete Ayele Desimmie, Caroline Weydert, Rik Schrijvers, Sofie Vets, Jonas Demeulemeester, Paul Proost, Igor Paron, Jan De Rijck, Jan Mast, Norbert Bannert, Rik Gijsbers, Frauke Christ, Zeger Debyser

**Affiliations:** 1 Department of Pharmaceutical and Pharmacological Sciences, KU Leuven, Laboratory for Molecular Virology and Gene Therapy, Louvain, Flanders Belgium; 2 KU Leuven, Laboratory of Molecular Immunology, Rega Institute, Louvain, Flanders Belgium; 3 Department of Proteomics and Signal Transduction, Max-Planck Institute of Biochemistry, 82152 Martinsried, Germany; 4 Veterinary and Agrochemical Research Centre CODA-CERVA, Brussels, Belgium; 5 Robert Koch Institute, Centre for HIV and Retrovirology, Berlin, Germany; 6 Present Address: Viral Mutation Section, HIV Drug Resistance Program, Center for Cancer Research, National Cancer Institute, Frederick, MD USA

## Correction to: Retrovirology 12:16 (2015) 10.1186/s12977-014-0134-4

Following publication of their article [1], the authors realized that honest splices in two figures were not indicated. These splices do not affect in any case the results but ought to have mentioned in the legend or indicated by white separators in the figure.

In Fig. 1c, Fractions 4–11 were run on a different gel than 1–3 because of limited slots per gel. The representation (empty lanes) does not affect the result.

**Fig. 1:**
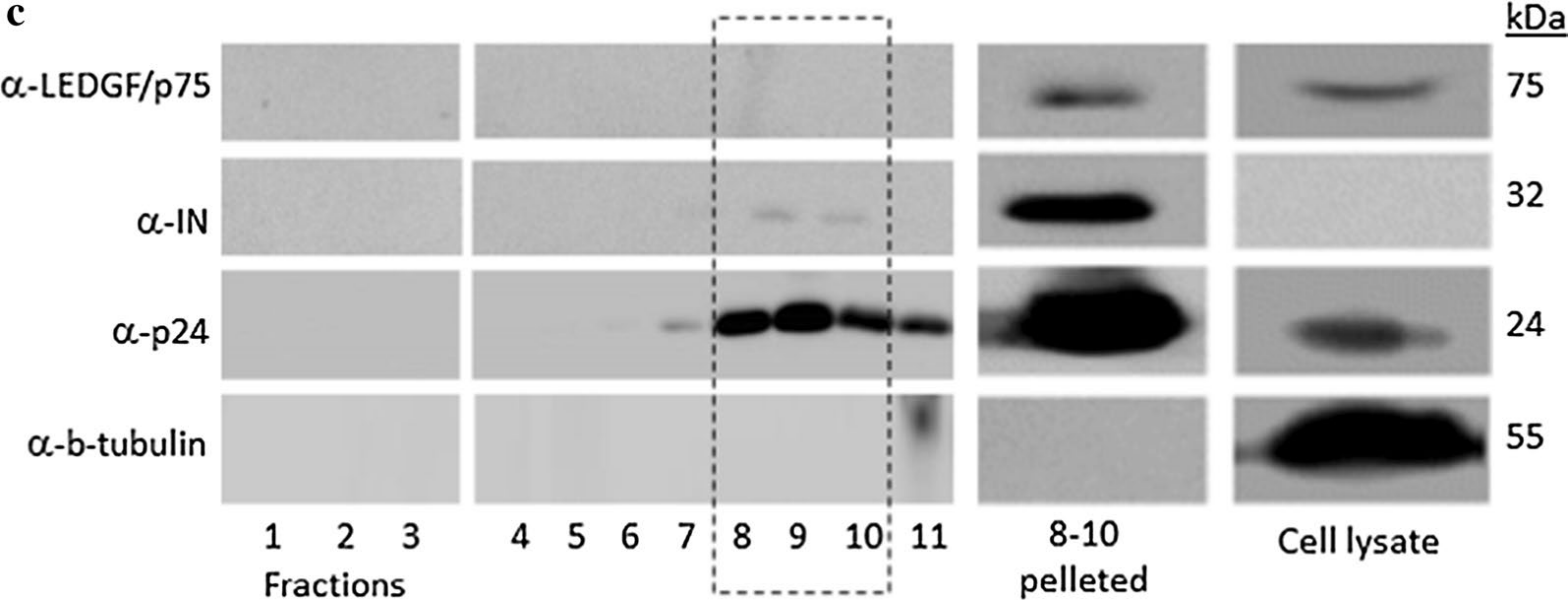
**c** Western blot analysis of individual fractions, pooled fractions 8–10 (delineated with a broken line) and producer cell lysate

In Fig. 4c we left out the MW marker but did not indicate the splice. Again, this representation did not affect the results.

**Fig. 4:**
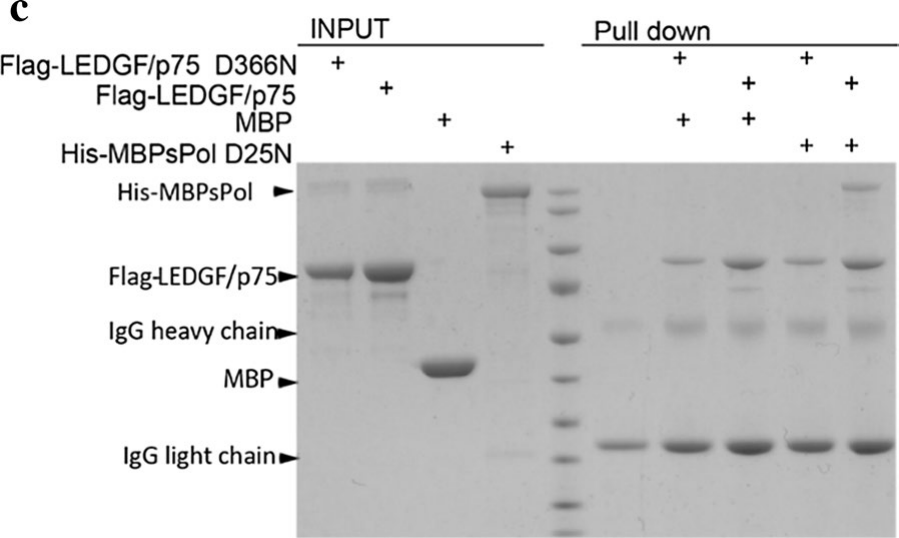
**c** Pull-down of His-MBP-sPol_PR_D25N_ using recombinant Flag-LEDGF/p75_WT_ or Flag-LEDGF/p75_D366N_. **A MW marker was run in between input and pull-down lanes**

Therefore, the authors would like to replace Figs. 1c and 4c with new ones, and update legends accordingly with the changes marked in bold.
