# Supplementary figures and images for: HIV-1 IN/Pol recruits LEDGF/p75 into viral particles
Source: Retrovirology. 2015 Feb 12;12:16. doi: 10.1186/s12977-014-0134-4 (PMC4357141; doi:10.1186/s12977-014-0134-4)

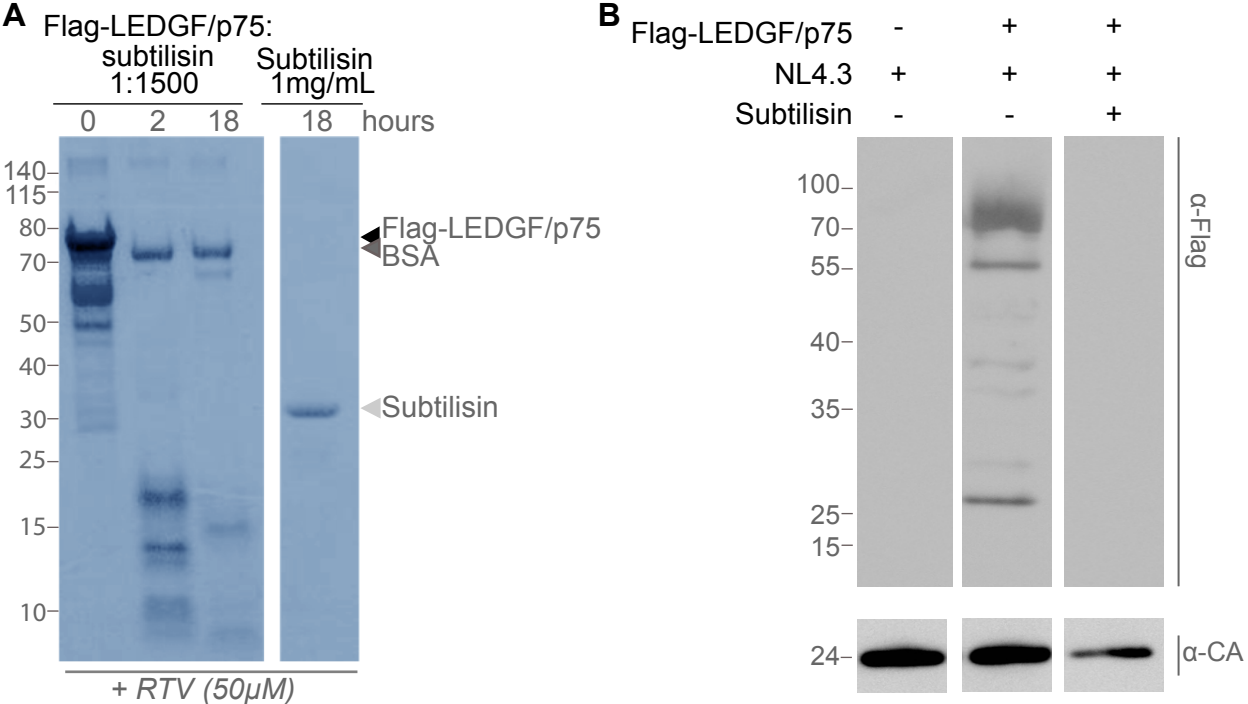

**Figure S1**

Supplement: Additional file 1: Figure S1. — Effect of subtilisin on LEDGF/p75 proteolysis. (A) Coomassie stained SDS-PAGE gel showing that LEDGF/p75 degradation by subtilisin is not inhibited by ritonavir (RTV). Lane 1-3: Limited proteolysis of LEDGF/p75 (20 μg) was performed at 37°C for different time intervals (0-2-18 hours) with 13.33 ng of subtilisin (1:1500 (w/w) ratio of enzyme to substrate). Lane 4: the proteolysis was performed for 18 hrs using 1 mg/ml subtilisin (enzyme to substrate ratio 1:0.8); this concentration was used during virus purification. Bands representing BSA and subtilisin are indicated with arrowheads. (B) Western blot analysis with anti-Flag antibody of subtilisin treated virus preparations spiked with recombinant Flag-LEDGF/p75. NL4.3 was spiked 4 hours with buffer (lane 1) or Flag-LEDGF/p75 (lane 2, 3) at 4°C and subsequently incubated at 37°C for 18 hours with (lane 3) or without (lane 1, 2) subtilisin. [file 12977_2014_134_MOESM1_ESM.pdf]

**A**    ●--- Mock AChE activity    ○--- Mock p24 ELISA  
          ■--- IIIB AChE activity    □--- IIIB p24 ELISA

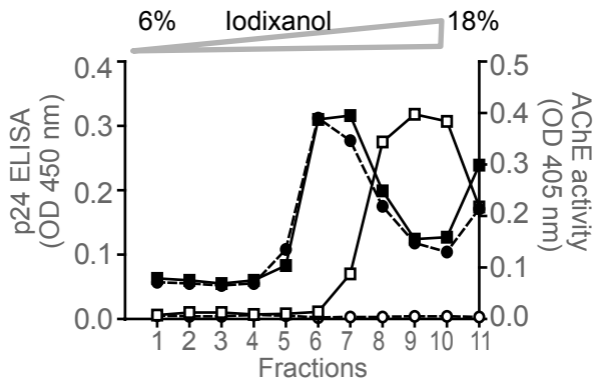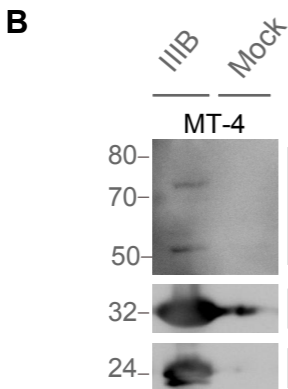

**Figure S2**

Supplement: Additional file 2: Figure S2. — Detection of LEDGF/p75 in purified virions produced in a T-cell line. Supernatant of HIV-1 IIIB (lane 1) and mock infected (lane 2) MT-4 cells was harvested, treated with subtilisin and purified over an iodixanol velocity gradient. (A) Acetylcholinesterase (AchE) activity and p24 content of the different fractions show an effective separation of exosomes and HIV virions. (B) Fractions 8-10 were pooled and subjected to Western blot analysis using anti-LEDGF/p75, anti-IN or anti-CA antibody. [file 12977_2014_134_MOESM2_ESM.pdf]

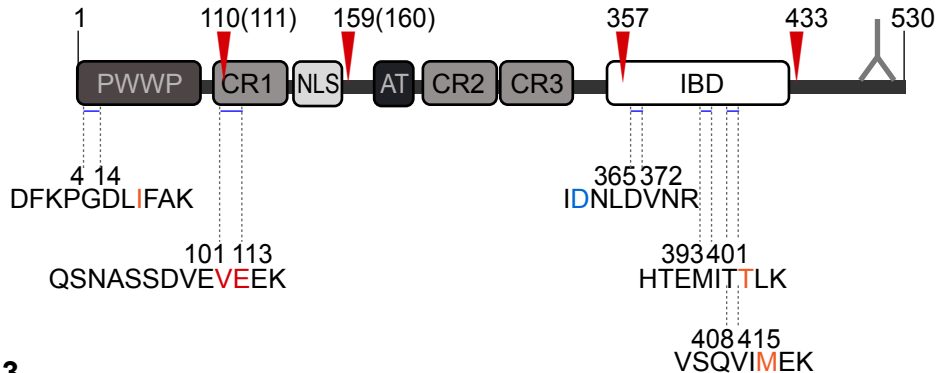

**Figure S3**

Supplement: Additional file 4: Figure S3. — LEDGF/p75 detected in purified HIV virions using Mass Spectrometry. Peptides described in Additional file 3: Table S1 mapped to LEDGF/p75. Confirmed (red) and predicted (orange) protease cleavage sites are indicated, as well as the integrase interacting D366 amino acid (blue). Protease cleavage sites were predicted with HIVcleave [38,55]. [file 12977_2014_134_MOESM4_ESM.pdf]

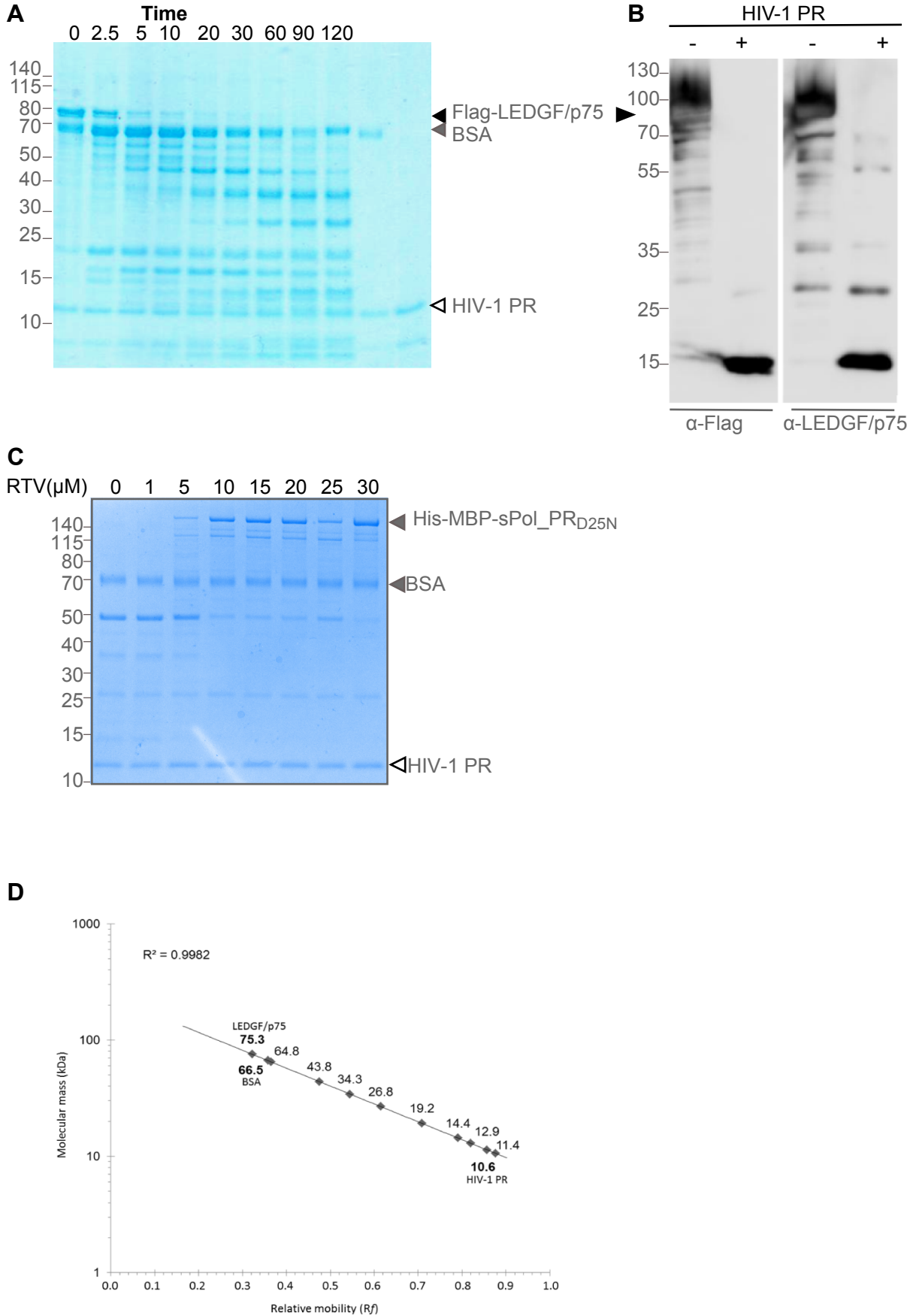

**Figure S4**

Supplement: Additional file 5: Figure S4. — In vitro limited proteolysis of LEDGF/p75 by HIV-1 PR and determination of molecular mass of cleavage products generated by HIV-1 PR. (A, B) HIV-1 PR cleavage products of pure recombinant Flag-LEDGF/p75 separated by SDS-PAGE. Coomassie brilliant blue staining (A) revealed the same cleavage pattern as untagged LEDGF/p75, while Western blot analysis (B) using anti-Flag antibody recognized N-terminal fragments or C-terminal fragments using anti-LEDGF/p75 antibody. Bands representing BSA and HIV-1 PR are indicated with arrowheads. (C) Cleavage of recombinant His-MBP-sPol_PRD25N by recombinant HIV-PR is inhibited by high concentrations of ritonavir (RTV). (D) Standard curve generated from the reference protein marker bands (R2 = 0.9982) as described before [1]. Molecular mass (kDa) of the different fragments of LEDGF/p75 and reference full-length proteins are indicated on the standard curve. [file 12977_2014_134_MOESM5_ESM.pdf]

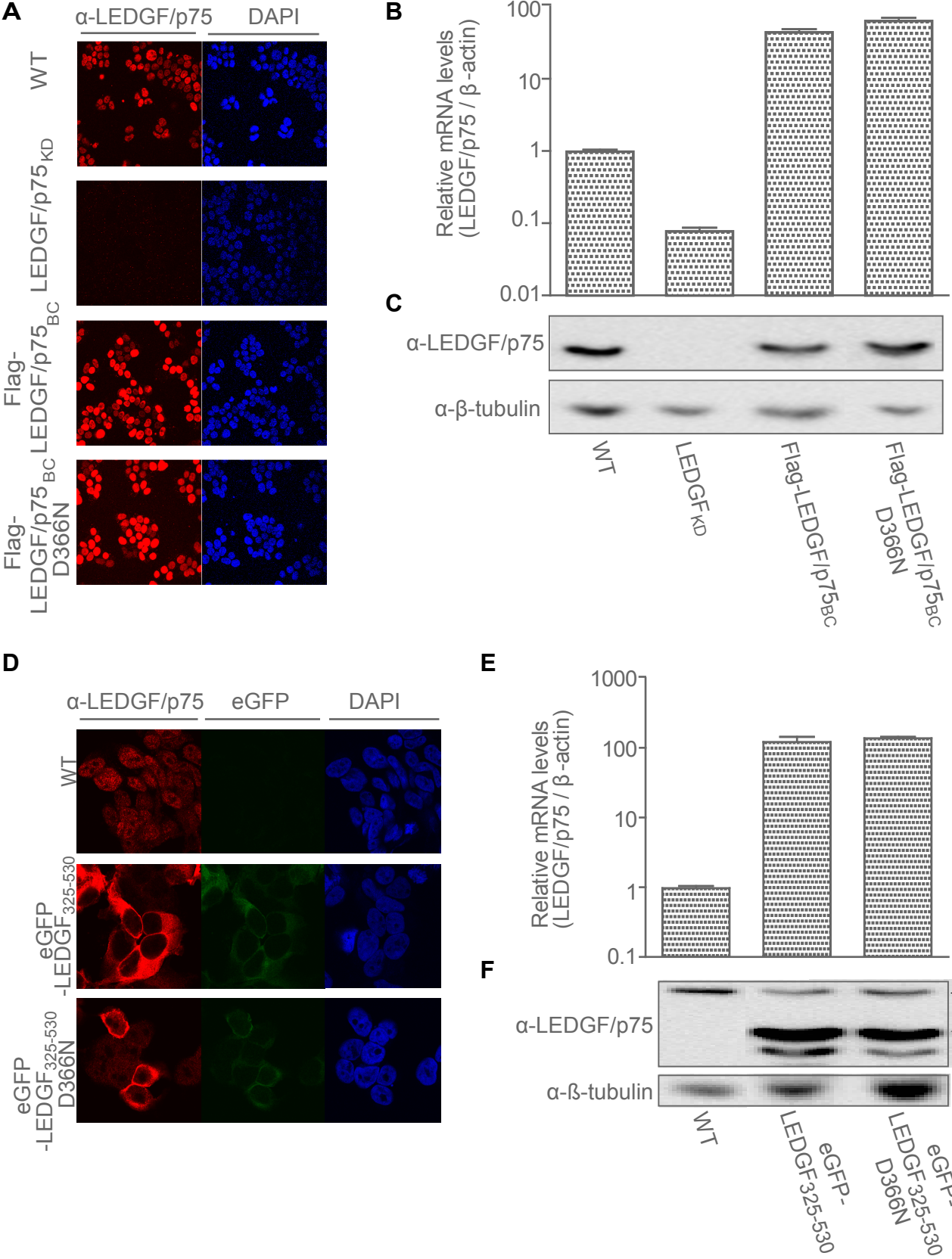

**Figure S5**

Supplement: Additional file 6: Figure S5. — Characterization of the expression of different LEDGF/p75 constructs. (A, B, C) LEDGF/p75-depleted cells (LEDGF/p75KD) were back-complemented with lentiviral vectors encoding either Flag-LEDGF/p75 (LEDGF/p75BC) or Flag-LEDGF/p75BCD366N. (A) Fluorescence microscopy images of cells stained with anti-LEDGF/75 antibody (red). Nuclei were stained with DAPI (blue). The levels of protein and mRNA for the different LEDGF/p75 constructs were determined by RT-qPCR (B) and Western blot analysis (C), respectively. β-tubulin was included as a loading control. (D, E, F) Stable overexpression of eGFP-LEDGF325-530 or eGFP-LEDGF325-530D366N in 293T cells verified with (D) fluorescence microscopy (anti-LEDGF/p75 antibody: red; eGFP: green; DAPI: blue), (E) RT-qPCR or (F) Western blotting (LEDGF/p75: black arrowhead, eGFP-LEDGF325-530: open arrowhead). [file 12977_2014_134_MOESM6_ESM.pdf]

**A**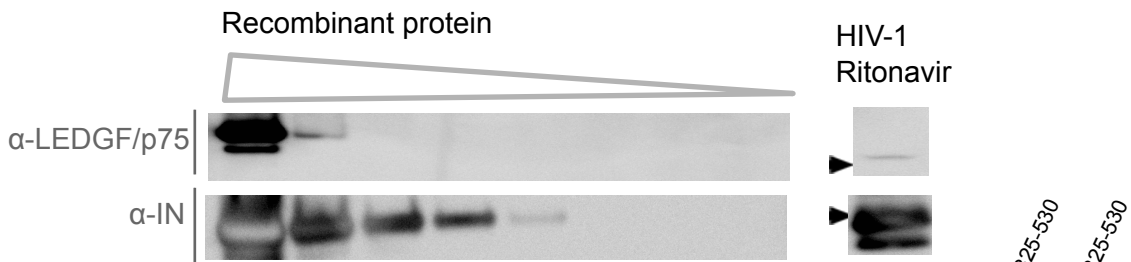**B**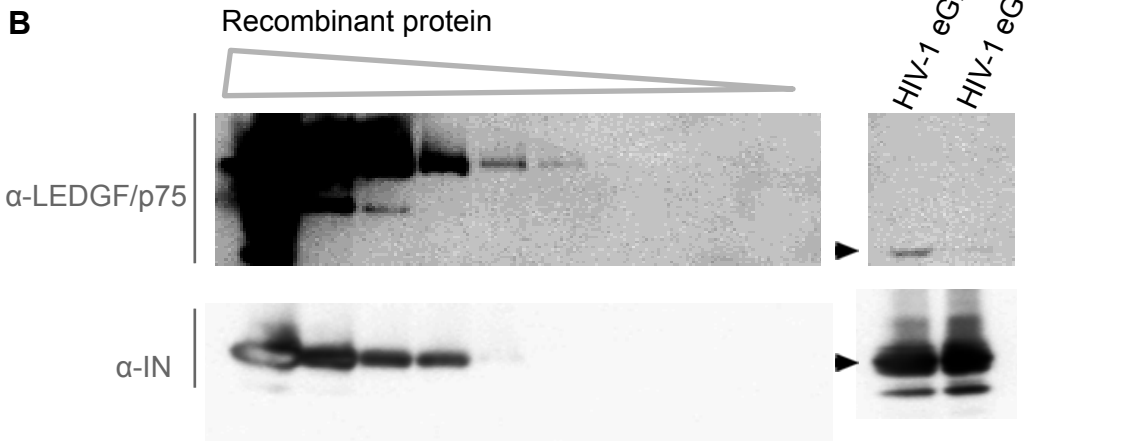**Figure S6**

Supplement: Additional file 7: Figure S6. — Quantification of LEDGF/p75 or eGFP-LEDGF325-530 in HIV particles. (A, B) Western blot of recombinant Flag-LEDGF/p75 (upper lanes) or His-IN (lower lanes) in a 1:3 dilution series, next to HIV virions harvested, subtilisin treated, purified and concentrated from WT 293T cells (A) or cells overexpressing eGFP-LEDGF325-530 or eGFP-LEDGF325-530D366N (B) using anti-LEDGF/p75 (upper lanes) or anti-IN (lower lanes) antibody. [file 12977_2014_134_MOESM7_ESM.pdf]
