# Supplementary material for: HIV-1 IN/Pol recruits LEDGF/p75 into viral particles
Source: Retrovirology. 2015 Feb 12;12:16. doi: 10.1186/s12977-014-0134-4 (PMC4357141; doi:10.1186/s12977-014-0134-4)
Supplement: Additional file 3 Table S1. — LEDGF/p75 peptides detected from purified HIV virions using Mass Spectrometry. Table S2. Determination of the molecular mass of LEDGF/p75 fragments generated by HIV-1 PR in vitro. Table S3. N-terminal sequencing of LEDGF/p75-derived fragments following HIV-1 PR digestion. Table S4. Identified cleavage site sequences and cleavage prediction algorithm of octapeptides derived from LEDGF/p75 cleavage by HIV-1 PR. [file 12977_2014_134_MOESM3_ESM.pdf]

Supporting Tables

Table S1. LEDGF/p75 (peptides) detected in purified HIV virions using Mass Spectrometry.

Gene name	Protein name	Sequence	In # samples found (4)
PSIP1	PC4 and SFRS1-interacting protein	DFKPGDLIFAK	4
		QSNASSDVEVEEK	1
		IDNLDVNR	2
		HTEMITTLK	2
		VSQVIMEK	4

HIV virions were obtained from the supernatant of 293T cells, purified through an iodixanol velocity gradient and treated with subtilisin. Lysed samples were run in SDS-page; part of the gel corresponding to proteins of 75 kDA was cut out, and proteins extracted subjected to mass spectrometry analysis. HIV samples were matched to HeLa samples which were processed in parallel.

Table S2. Determination of the molecular mass of LEDGF/p75 fragments generated by HIV-1 PR *in vitro*

STANDARD CURVE			Flag-LEDGF/p75 fragments	
R_f (mm)	Marker MW (kDa)	Calulated MW (kDa)	R_f (mm)	Calulated MW (kDa)
0.164	140	137		
0.213	115	115	0.311	75.3*
0.300	80	84	0.365	64.8
0.343	70	73	0.475	43.8
0.430	50	53	0.545	34.3
0.496	40	42	0.615	26.8
0.586	30	30	0.709	19.2
0.652	25	24	0.791	14.4
0.766	15	16	0.820	12.9
0.920	10	10	0.857	11.4
Reference protein	HIV-1 PR		0.877	10.6
	BSA		0.358	66.5

Calculations based on relative mobility (Rf) as shown in Figures 2 and S3c and S. HIV-1 PR and BSA were used as reference proteins. *Calculated molecular mass of full length LEDGF/p75

Table S3. N-terminal sequencing of LEDGF/p75-derived fragments following HIV-1 PR digestion

Cycle no.	Prime-side (P') residues [a]	HIV-1 PR cleavage products of LEDGF/p75			
		**43.8 kDa**	**34.3 kDa**	**26.8 kDa**	**10.6-12.9 kDa (overlapping bands)** [b]
1	P_1'	V,E,T	E,T,V	E,T	D,A,**M**,P
2	P_2'	E	E,T	E	S,**T**,E,Q
3	P_3'	E	A,E	A,E	V,I,**R**,L
4	P_4'	T	G,T	G,A	T,E,A,V
5	P_5'	T,E	V,T,E	V	L,G,V
6	P_6'	S,T	V,T	V	Q,T,E
7	P_7'	S	T	T	
8	P_8'	V	T	T	
9	P_9'		A,E		
10	P_{10}'		T,A		
11	P_{11}'		A,T		
12	P_{12}'		S		

LEDGF/p75 was treated with HIV-1 PR for 2 h at 37°C as described in the Extended Experimental Procedures section. [a] Residues C-terminal to the scissile peptide bond of the cleavage sites are designated as prime-side (P') [1]. Fragment 43.8, 34.3, 26.8, and overlapping bands (10.6, 11.4 and 12.9 kDa) were sequenced by N-terminal protein sequencing and the prime-side (P') amino acids identified after each cycle of Edman degradation are listed. [b] Four possible sequences were identified representing the N-terminal amino acids of LEDGF/p75 (bold) and HIV-1 PR (red), as well as two PR derived LEDGF/p75 cleavage products.

Table S4. Identified cleavage site sequences and cleavage prediction algorithm of octapeptides derived from LEDGF/p75 cleavage by HIV-1 PR.

Enzyme	Cleavage site in LEDGF/p75 [a, b]	Predicted algorithm Δ27[c]
HIV- PR	SDVE↓V^{110}EEK [d]	0.81
	DVEV↓E^{111}EKE	0.85
	KQVE↓T^{159}EEA	1.20
	QVET↓E^{160}EAG	1.01
	QRIH↓A^{357}EIK	0.80
	VGEG↓D^{433}SVI	0.70
Caspase-3 and -7 [e]	DEVPD^{30}↓G	-
	WEID^{85}↓N	-
	DAQD^{486}↓G	-

[a] Arrow indicates the scissile peptide bond of sequenced cleavage sites of the indicated enzyme. [b] The predicted cleavage site oligopeptide sequences confirmed by N-terminal sequencing. [c] Determined using the HIVcleave prediction program[1, 2]. [d] The underlined residues with the superscript numbers represent the C-terminal (prime-side; P') to the scissile peptide bond of the cleavage sites of HIV-1 PR, and caspase-3 and -7, respectively. [e] Caspase-3 and -7 cleavage site sequences in LEDGF/p75 described in [2].

Supporting References

1 Schechter I, Berger A: **On the size of the active site in proteases. I. Papain.** *Biochemical and Biophysical Research Communications* 1967, **27:**157-162.

2 Wu X, Daniels T, Molinaro C, Lilly MB, Casiano CA: **Caspase cleavage of the nuclear autoantigen LEDGF/p75 abrogates its pro-survival function: implications for autoimmunity in atopic disorders.** *Cell Death Differ* 2002, **9:**915-925.
